# Breakthroughs and Applications of Organ-on-a-Chip Technology

**DOI:** 10.3390/cells11111828

**Published:** 2022-06-02

**Authors:** Mufeeda C. Koyilot, Priyadarshini Natarajan, Clayton R. Hunt, Sonish Sivarajkumar, Romy Roy, Shreeram Joglekar, Shruti Pandita, Carl W. Tong, Shamsudheen Marakkar, Lakshminarayanan Subramanian, Shalini S. Yadav, Anoop V. Cherian, Tej K. Pandita, Khader Shameer, Kamlesh K. Yadav

**Affiliations:** 1 Molecular Robotics, Cochin 682033, India; mufeedack.mec@gmail.com (M.C.K.); darshinatarajan@gmail.com (P.N.); sonish.sivarajkumar@gmail.com (S.S.); romyroyz@gmail.com (R.R.); shreeramjoglekar@gmail.com (S.J.); shamsudheenmarakkar17@gmail.com (S.M.); anoopvadakan@gmail.com (A.V.C.); 2 Houston Methodist Research Institute, Houston, TX 77030, USA; crhunt305@gmail.com; 3 Mays Cancer Center, University of Texas Health Sciences Center at San Antonio, San Antonio, TX 78229, USA; pandita@uthscsa.edu; 4 School of Engineering Medicine, Texas A&M University, Houston, TX 77030, USA; ctong@tamu.edu; 5 Courant Institute of Mathematical Science, New York University, New York, NY 10012, USA; Lakshmi@nyu.edu; 6 Department of Immunology, UT MD Anderson Cancer Center, Houston, TX 77030, USA; ssingh8@mdanderson.org; 7 Center for Genomic and Precision Medicine, Institute of Biosciences and Technology, Department of Translational Medical Sciences, Texas A&M University, Houston, TX 77030, USA; 8 School of Public Health, Faculty of Medicine, Imperial College London, South Kensington, London SW7 2AZ, UK

**Keywords:** organ-on-a-chip, heart, kidney, lung, liver, technology

## Abstract

Organ-on-a-chip (OOAC) is an emerging technology based on microfluid platforms and in vitro cell culture that has a promising future in the healthcare industry. The numerous advantages of OOAC over conventional systems make it highly popular. The chip is an innovative combination of novel technologies, including lab-on-a-chip, microfluidics, biomaterials, and tissue engineering. This paper begins by analyzing the need for the development of OOAC followed by a brief introduction to the technology. Later sections discuss and review the various types of OOACs and the fabrication materials used. The implementation of artificial intelligence in the system makes it more advanced, thereby helping to provide a more accurate diagnosis as well as convenient data management. We introduce selected OOAC projects, including applications to organ/disease modelling, pharmacology, personalized medicine, and dentistry. Finally, we point out certain challenges that need to be surmounted in order to further develop and upgrade the current systems.

## 1. Introduction

Animals such as mice, pigs, and sheep have been commonly used for experimentation, testing, teaching, and surgery. According to the United States Department of Agriculture, around 780,070 animals were utilized in the United States during the year 2018. However, the results generated from these animal models are often not confirmed in human clinical studies [1]. This has spurred the development of in vitro study methods focused on human cells, such as 2D and 3D cell cultures, but these processes are also unreliable, failing to mimic the complex and dynamic operations occurring in a human body [2]. Drug development involves extensive preclinical and clinical testing to validate toxicity and efficacy. Issues at any developmental stage can incur huge financial costs to pharmaceutical companies, a factor underlying the decline in the number of new drugs approved each year [3]. Drug-induced toxicity is one of the most important issues resulting from inaccurate preclinical prediction models; for example, ~20% of acute kidney injuries (AKI) after hospitalization are caused by drug-induced nephrotoxicity [4]. Thus, there is a critical need for more accurate and precise technology to recreate the therapeutically relevant features of an organ in relation to interventional drugs, including examining drug delivery and real-time monitoring of the cell and tissue response to a particular drug or infection.

**Figure 1:**
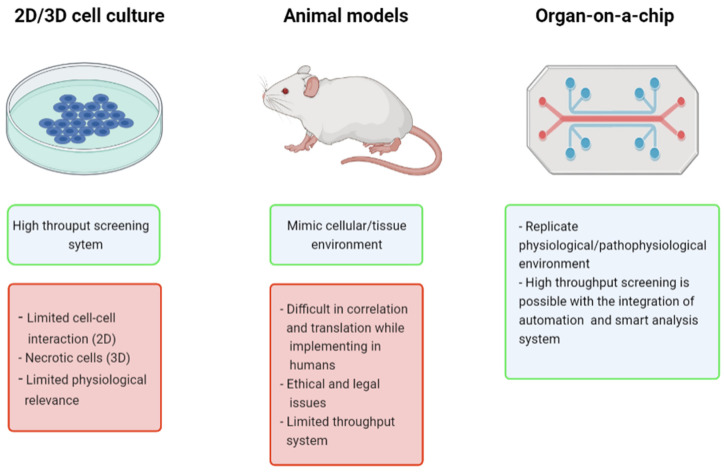
Some of the advantages of OOAC technology over cell cultures and animal models. Figure generated using BioRender.com (accessed on 16 May 2022).

Implementing the principles of microfluidics, tissue engineering, and lab-on-a-chip (LOC) technologies together has given rise to the emerging technique of organ-on-a-chip (OOAC), which utilizes miniaturized cell-culturing microenvironments with microchannels and chambers that replicate human cells’ natural environment [5]. Some of the advantages of the OOAC platform over conventional methods are outlined in Figure 1. A biocompatible material, polydimethylsiloxane (PDMS), is mainly used for fabrication given its excellent transparency and elasticity, but it has poor chemical resistance as it absorbs certain organic compounds, drugs, and biomolecules [6,7]. Other biocompatible materials that can be conveniently used for fabrication are being developed, including polymethylmethacrylate (PMMA), a cheaper but more rigid material than PDMS. Substances such as polystyrene (PS), polycarbonate (PC), polyimide (PI), collagen, gelatin, and alginate have also been utilized for fabrication [8,9]; however, these have limitations when considering individual properties such as elasticity, transparency, and chemical resistance, which must be accounted for when choosing the right material. Given the modular nature of OOAC, multiple modules such as actuators and sensors can be incorporated for various analyses. Compared to other existing methods, they are more precise and provide highly relevant clinical data. Moreover, OOAC can accommodate multiple cell layers, which mimic the complex cell interactions that occur in tissues. Multiple organs can also be connected, enabling the simultaneous analysis of different organs. The advancement in 3D bioprinting and 3D microfabrication that help in tissue modelling and preparation of organ-like structures have catalyzed the growth of OOAC technologies, with a wide range of applications, such as personalized medicine, micro-robotics, drug delivery, therapeutics, and many more. Since micro physiological in vitro modelling depends on several factors (including microfluidics, chip engineering, biomarkers, biomaterials, and the cell source), advancement in OOAC technology depends on the development of all these fields. Biological cell sources have a vital role in OOAC development, including induced pluripotent stem cell (iPSCs), adult stem cell (ASC), and embryonic stem cells (ESCs) [10]. iPSCs are commonly used for OOAC development since they can be obtained from a person with a specific disease phenotype. Additionally, their ability to differentiate and their biomimetic properties are far superior to other stem cell sources [11]. ESCs have many ethical controversies and regulatory issues associated with them as they are obtained from a human embryo [12]. ASCs are not widely used since they lack consistency in the derivation protocol. The advances in stem cell biology and stem cell engineering can contribute a promising future to OOAC technology.

Since pathological abnormalities are rarely the result of expression changes in single genes, gene chips have been generated to study comprehensive expression profiles, a critical step forward in elucidating normal biology and disease processes. Perusal of the literature has revealed the utility of the gene chip for the analysis of the impact of a single gene on the expression of multiple genes [13,14]. Examples of emerging OOAC technologies include heart-on-a-chip, lung-on-a-chip, kidney-on-a-chip, bone-on-a-chip, liver-on-a-chip, and skin-on-a-chip. In addition, the burgeoning of personalized precision medicine has contributed to the development of cancer-on-a-chip and in vitro models to monitor conditions such as non-alcoholic fatty liver disease (NAFLD). When multiple organs or complex tissues are integrated on a chip, a large amount of data is produced, which requires high-throughput analyses to reach accurate conclusions. Artificial intelligence (AI), mainly machine learning techniques, is one of the most reliable methods to train the analytics for predicting or classifying the output based on similar data sets. Integration of OOAC and AI has the potential to remove barriers that limit applications directly relevant to human health, while overcoming ethical approval issues [15]. Incorporating imaging devices, such as time-lapse microscopy, into OOAC technology helps to obtain high-quality videos for analysis [16]. The captured videos combined with machine learning algorithms provide enhanced monitoring of the underlying processes, which is a promising approach for pharmacology and drug discovery tasks.

From this perspective, we have examined the different types of OOAC platforms and their application including an overall discussion of the major advancements in certain chip platforms. We have discussed different fabrication techniques involved in design and development, such as those developed to replicate the anatomical and physiological structure and function of organs, and models for diseases associated with certain organs, along with some of the pros and cons of these platforms. It is highly evident that OOAC technology has major potential in the healthcare sector and integration of multiple recent technologies will help to develop a highly advanced system. Hence, we emphasized the need for integrating AI into these systems, identified crucial government-supported projects in OOAC, and the leading manufacturers that are contributing to developing new OOAC technologies. Potential barriers that need to be carefully addressed before the technology can be transferred from the laboratory to the clinical market are also mentioned. We conclude with the challenges facing these platforms that need to be addressed to develop a more reliable and accurate system.

## 2. Types of OOAC Devices

### 2.1. Lung-on-a-Chip

**Figure 2:**
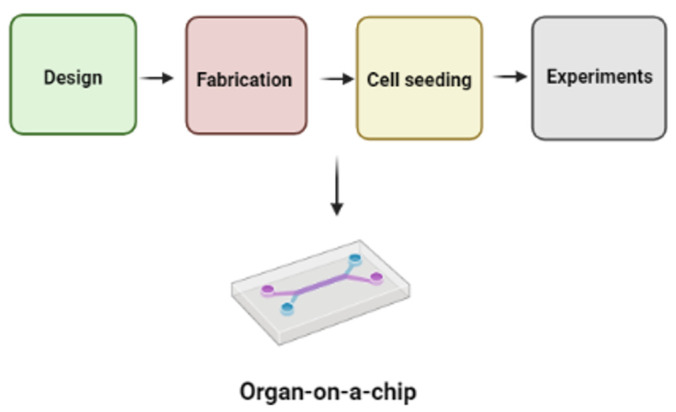
Example of processes involved in developing OOAC devices. Figure generated using BioRender.com (accessed on 16 May 2022).

According to the World Health Organization (WHO), pulmonary diseases were the third leading cause of death in 2019, suggesting adequate treatment options are lacking. In addition, the COVID-19 outbreak has amplified the need for pulmonological research due to the associated high levels of lung damage. A simple representation of procedures needed to develop an OOAC platform (illustrated in Figure 2) suggests that different techniques can be adapted for fabricating lung-on-a-chip devices (Table 1). Huh and co-workers [17] designed pulmonary edema-on-a-chip, which is a PDMS-based device fabricated using soft lithography techniques. The device is fabricated in such a way that there are upper and lower microchannels with an extracellular matrix (ECM) in between them, while the side chambers were created by etching the membrane layers. After sterilization, cultured alveolar cells are seeded in the upper channel whereas endothelial cells occupy the opposite channel. The alveolar-capillary barrier formed is stretched to replicate the breathing mechanism. Though beneficial for drug and toxicity testing, the device cannot fully emulate organ-level lung functions. Humayun et al. [18] developed a lung airway-on-a-chip to investigate the interaction between smooth muscle cells (SMCs) and epithelial cells (ECs). The device was fabricated from PMMA using micromilling and solvent bonding techniques. It consists of two compartments at the top and two at the bottom, with different airway cell types and a hydrogel medium in the middle chamber. Cell-laden hydrogel extraction is possible with this device without cross-contamination. Another 3D cell bioprinted airway-on-a-chip model was constructed by Park et al. [19] to replicate the interface between the airway epithelium and the vascular network. In this case, a 3D microvascular network was initially formed using endothelial cell bioink, lung fibroblast bioink, polycaprolactone (PCL), and PDMS. PCL was printed depending on the purpose, bioinks were injected to their specific location, and PDMS was shot into the frame. This platform was found to be useful in preclinical drug testing and screening. Recently, Bai et al. were able to demonstrate physiological correlation between the mechanical breathing motions of lung tissues with host innate immunity using a human lung alveolus chip that experiences cyclic breathing-like deformations [20]. This marked a major advancement in the field when compared with static OOAC platforms as it revealed that breathing motions could suppress viral replication by activating protective innate immune responses in epithelial and endothelial cells [20].

### 2.2. Heart-on-a-Chip

Cardiovascular diseases (CVD) are a leading cause of death worldwide [21]. WHO estimates that by 2030 around 23.6 million lives will be lost to CVD. Drug-induced cardio toxicities are one of the primary reasons for drug withdrawal post market introduction; in fact, high drug attrition rates are a significant challenge facing the pharmaceutical industry [22,23,24]. The use of heart-on-a-chip to study the functions and interactions of heart muscles as well as pharmaceutical testing can contribute to the development of effective therapeutic mechanisms for CVDs. However, a significant challenge is the limited potential for proliferation of mature cardiomyocytes, which prevents the reproduction of injured/diseased hearts [25]. Recent studies, therefore, have focused on enhancing proliferation by modulating cell cycle regulators [26,27,28].

Several techniques, such as soft lithography, laser-based methods, electrospinning, and 3D bioprinting, have been employed to fabricate heart-on-a-chip. In in vitro cardiovascular models, under static conditions, tissues do not exhibit orientations, alignments, or flow stipulations, thus fail to recapitulate the native heart. Jain and co-workers [29] engineered cardiomyocytes with orientations and alignment that emulate the heart’s cellular microenvironment. Micro-ridges on silicon wafers were fabricated using optical lithographic techniques, followed by reactive ion etching. The model was mainly intended to study cardiac hypertrophy and the associated intrinsic alignment of tissues provided a favorable condition for the fast conduction of ions. Marsano et al. [30] developed a heart-on-a-chip platform to produce highly functional cardiac tissue from neonatal rats and human iPSCs-derived cardiomyocytes. They adopted soft lithographic techniques for its robustness to fabricate the device, and PDMS due to its elastic and optical properties. This setup enabled mature and functional 3D cardiac micro-tissues and the study of their interactions after supplementation with a particular drug. A high throughput heart-on-a-chip was developed by Agarwal and co-workers [31], which was used to accurately analyze the contractile response of muscular engineered tissue. The system could also be used to identify interactions with various cardiac drugs. Here, laser-based techniques and computer-aided designs were adopted with PDMS as the material for fabrication. Since the developed design offers high throughput with optimal reproducibility, it could eliminate translation barriers arising during commercialization. With the help of electrospinning and crosslinking techniques, human iPSCs could be differentiated into a functional cardiomyocyte layer producing homogeneous contractions. This developed layer could be subsequently used to analyze drug interactions and responses with heart tissues [32]. 3D bioprinting has been used by Zhang et al. [33] to produce myocardial tissues and combine them with bioreactors so they could be used for cardiovascular drug screening.

Bioelectronic technologies have also been employed for heart-on-a-chip to record physical and electrical measurements of cardiac functionalities. Such biosensors, in the form of carbon nanotubes, were utilized to sense contractility and action potentials. Wang et al. [34] utilized these models to test and validate drugs, as these sensors are highly flexible and have excellent resistance-measurement capabilities. A 3D soft electrode array has been used to record cardiomyocyte electrophysiology because of its flexibility and sensing of heart muscle deflection during contraction and relaxation [35]. A recently engineered bioelectronic-based heart-on-a-chip by Liu and co-workers [36] assessed electrophysiological response to hypoxia. The device was fabricated with photolithography techniques. Planar electrodes were employed for extracellular measurements, while nanopillar arrays were used for intracellular measurements. The fluidic device was prepared from biocompatible PDMS by linking them with the prepared bioelectronic chip, into which HL-1 cardiac muscle cells were seeded. With the incorporated electrode, the authors observed patterns associated with hypoxia-induced tachycardia. With nanopillar electrodes, intracellular measurements of a single-cell-based action potential was obtained. The development of a fully functional biosensor-based heart-on-a-chip device would help in real-time analyses of heart functions upon their stimulation with substances, such as drugs and hormones, and thus would be an invaluable tool in cardiovascular studies.

### 2.3. Kidney-on-a-Chip

The human kidney is responsible for maintaining homeostasis associated with the removal of unwanted or toxic substances, and reabsorption of substances required for the proper functioning of the body; drug and toxin-induced kidney damage can lead to permanent renal failure. The role of drug-induced nephrotoxicity in acute kidney injury (AKI) indicates the relevance of accurate drug testing and screening systems. However, successful preclinical animal trials cannot be directly applied to the human body because of the differences in metabolic mechanisms.

The functional kidney unit, the nephron, consists of renal corpuscles and renal tubules where filtration and reabsorption occur. Even though the initial experimental device to observe the tubular flow of renal cells was designed in 2001 by Essig and co-workers, [37] a breakthrough design for kidney-on-a-chip was proposed by Weinberg and co-workers [38] in 2008. They developed a bioartificial device based on a micro-electro-mechanical system (MEMS). It was assembled through microfabrication techniques and had the ability of diffusion, thus mimicking the property of Henle’s loop, an integral part of the kidney filtration system. Designs for glomerulus and proximal tubules have also been developed along with Henle’s loop, which together can perform filtration and reabsorption as carried out by nephrons, though not as efficiently.

**Figure 3:**
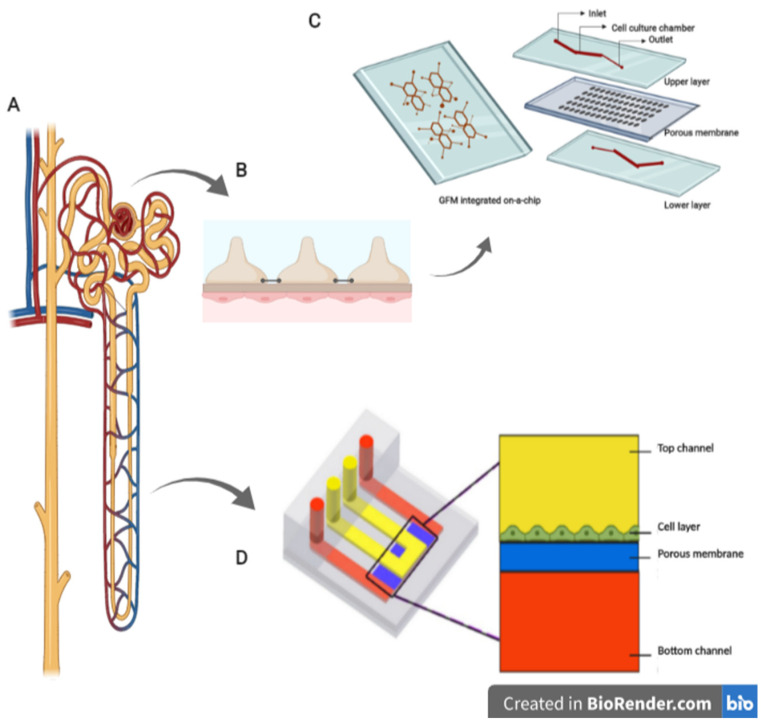
(**A**) Nephron with blood vessels. (**B**) Structure of glomerular filtration barrier, comprising endothelial cells, basement membrane, and podocytes. (**C**) Four glomerular filtration units incorporated on a single chip to form glomerular filtration membrane (GFM). The figure is referred from Ref. [43]. Copyright © 2016, Zhou et al. (**D**) Schematic representation of tubule on a chip. The figure is modified from Ref. [41]. Copyright © 2016, Kim et al. The diagram was generated using BioRender.com (accessed on 16 May 2022).

To recreate the functions of renal tubular cells, Jang and co-workers [39] prepared a multilayer microfluidic device by culturing primary rat inner medullary duct cells (IMCD). A fluidic shear was applied to recreate an in vivo-like tubular environment for the cultured cells, and indeed the device could simulate the renal tubule system. Later, a modified version of this device with human kidney epithelial cells was developed where researchers compared static and fluidic culture conditions and concluded that physiological flow plays a vital role in maintaining the function of proximal tubule epithelial cells [40]. This model appeared to be applicable to renal toxicity testing, quantitative analysis of biological processes in kidney tubules, and in kidney pharmacology. To test for kidney toxicity, Kim et al. [41] fabricated a tubule-on-a-chip from PDMS (Figure 3D), where they cultured Madin-Darby canine kidney cells in the membrane and tested toxicity with the drug gentamicin.

Despite the above advancements, developing a glomerulus-on-a-chip has been challenging as it is difficult to reproduce the filtration barriers and culture podocytes. Yang et al. [42] developed a method using retinoic acid (RA) and fluidic shear stress (FSS) to replicate the in vivo environment of the glomerulus. The method improves podocyte differentiation and was useful in pathophysiological studies of glomerular diseases, diagnostics, and therapeutics for chronic kidney diseases. A functional glomerulus was engineered on a chip to assess hypertensive nephropathy by Zhou et al. [43]. To replicate the glomerulus, a basement membrane extract was coated on one side of the membrane, whereas glomerular endothelial cells and podocytes from mice were cultured on the other side of the membrane. A hypertensive environment was recreated by controlling the flow rate of the culture medium perfused through the membranes. To increase the efficiency of the experiment, sixteen culture chambers were developed in a single chip with a common inlet (Figure 3C).

### 2.4. Liver-on-a-Chip

The liver, the largest solid organ, is responsible for xenobiotic metabolism in addition to other physiological functions. Because of the poor efficiency of in vitro and animal models, toxicity studies are limited, which in turn delays drug development processes. According to the WHO, around 257 million people were affected with chronic hepatitis B infection and 887,000 deaths occurred during the year 2015. Although a preventative vaccine is available, post-infection treatments are still needed. Moreover, almost 44,000 people in the US suffer from drug-induced liver infection (DILI) [44]. Thus, development of a liver-on-a-chip device would have key applications in numerous diagnostic and therapeutic areas, particularly in developing novel therapeutics for chronic diseases, such as hepatitis B [45,46]^,^, liver cirrhosis [47], and hepatocellular carcinoma [48].

Hepatocytes are responsible for the main cellular functions in the liver. A significant challenge in developing liver-on-a-chip devices is to maintain hepatocyte functionality in an in vitro environment. Kane and co-workers [49] described a system with microfluidic arrays in which rat hepatocytes were co-cultured with 3T3-J2 fibroblasts and continuously suffused with cell culture medium and oxygen. The arrays were constructed by combining the principles of microlithography and soft lithography in multiple phases. With the perfusion of medium and oxygen, they observed the production of albumin and urea, thereby indicating the functional capacities of hepatocytes in the microfluidic array.

To improve liver-specific functionalities, a perfusion bioreactor was created to obtain spheroids, as these have a crucial role in long-term studies related to liver metabolism [50]. Spheroids cultured in an automated bioreactor offer a liver-like environment and liver-specific functionalities. A spheroid-based three-dimensional (3D) liver culture model was built by combining PDMS techniques, which resulted in the contriving of V-shaped concave microwell arrays and enabled formation of uniform-sized spheroids. This system allows for convenient and rapid perfusion of hepatic spheroids [51].

Despite several efforts, a liver-on-a-chip device that completely replicates human liver function, such as ECM for bile secretion, is still lacking [52]. Recently, Lee et al. [53] generated a system that overcomes most of these limitations using cell printing technology and biomaterials to form a 3D liver-on-a-chip. The performance of this system in terms of ECM and the biliary fluidic channel is superior to previous 2D/3D culture systems, thereby opening the door to testing diverse therapeutic, diagnostic, and pharmaceutical processes.

The OOAC technology has indeed come a long way, but there still exist some limitations which will need to be worked upon to make it an in vivo alternative. Towards this, a detailed comparison of the advantages and disadvantages of the various types of OOAC discussed in the text is presented in Table 2.

### 2.5. Other OOAC Devices

Developments in chip engineering, cell biology, and microfluidics have accelerated the progress of different types of organs and organ systems on a chip. One of the limitations of the OOAC technology is the generation of physiologically relevant oxygen concentrations in the system. Recently, Grant et al. described a simple strategy to achieve a sustained and physiologically relevant oxygen concentration by growing cells in specific locations and allowing them to decrease the oxygen concentration through aerobic respiration. In this way, they were able to generate a consistent oxygen gradient in systems cultured in a conventional aerobic cell culture incubator [59]. Cosmetic products often cause allergic and inflammatory reactions in certain people by activating the immune system. The availability of skin-on-a-chip devices would allow for cosmetic testing to investigate inflammation, psoriasis, and other skin-related pathologies [60]. When developing skin-on-a-chip models, it is necessary to reconstitute all the relevant parts, i.e., epidermis, basal cell layer, dermis, and hypodermis. Techniques such as lithography [61,62,63], micromilling [64], laser cutting [65], and 3D printing [66] have been utilized to fabricate skin-on-a-chip platforms. However, there is no fully integrated platform yet that completely mimics the structural and functional properties of the skin.

Bone-on-a-chip technology can provide physiological and pathophysiological models for bone studies relevant to hematopoietic stem cell niche [67], prosthetic organs, bioimplants, and for assessing conditions such as osteoporosis, bone metastasis, and other bone-related issues. Tang et al. [68] produced a microfluidic device fabricated from hydroxyapatite and PDMS. This chip can be utilized to mimic a bone-like environment, thus being helpful in drug screening and other therapeutic applications.

## 3. Role of Artificial Intelligence in OOAC

Different OOAC systems produce a large amount of data that must be carefully evaluated for accurate interpretation. The integration of multiple OOAC devices into a single platform, especially in cancer and metastatic studies for various analyses, results in a high throughput system that generates unprecedented amounts of data [69,70]. For example, an experimental study on gut-on-a-chip incorporating 357 gut tubes generated 20,000 data points, which is the largest known OOAC dataset to date [71]. The use of cancer-on-a-chip or body-on-a-chip devices will generate a massive data flow and prolonged data collection that will require data management and complex analyses [72]. To this end, AI and big data analytics advancements can offer a reliable resource to deal with the generated information, both for storage and for analyses.

Machine learning algorithms can be applied to analyze and classify data. There are three main types of approaches: supervised learning, unsupervised learning, and reinforcement learning. Labelled data are added in the former, while unlabeled data are provided in the unsupervised learning method. Before adopting an algorithm, its features and applications must be considered. Other factors, such as data size, and whether the predictions and classifications are from an existing dataset or real-time analysis, etc., also need to be considered. These algorithms consider features and parameters depending on their purposes. For example, parameters such as number of cells, number of samples, and attributes such as solubility and oxygen concentration were considered during the screening of oncogenic viruses. When a certain amount of data is obtained from the observed parameters, it is divided into two parts: a training dataset for modelling and another dataset for validation. Machine learning algorithms are used to train the dataset and develop a model which predicts subsequent new data. Cross-validation can be conducted to evaluate the performance of the system [73].

AI can be integrated with the disease on a chip to provide more affordable and advanced diagnostic and therapeutic approaches. Kongadzem and co-workers explained their work on machine learning approaches in integrated organ-on-a-chip to evaluate drug efficacy. Support vector machine learning algorithms have been used to predict drug effectiveness and it has been observed that classification error is least when the sample data is smaller than the training dataset. Larsen et al. [74] collected samples from 1000 cancer patients to develop a tumor organoid platform to generate an artificial neural network-based therapeutic profiling assay for pan cancer analysis using label-free light microscopic images.

Although the application of AI helps to increase the performance of OOAC devices, the ability and efficiency of machine learning approaches depends on the availability of an adequate amount of data. Poor data with excessive noise and insufficient sampling can adversely affect the predicted output. However, the multiple approaches being investigated to model diseases on chips for molecular diagnosis and immunotherapy are likely to overcome these problems and power revolutionary developments in healthcare.

## 4. OOAC Platforms

OOAC is an evolving technology in the early stages of development, but its applications suggest a promising future. The current COVID-19 outbreak has amplified the research and development of OOAC, especially for lung-on-a-chip devices [75,76]. Hence, these devices are expected to experience unprecedented growth and deliver a boost in Compound Annual Growth Rate (CAGR) in the forthcoming years. Within the last decade, several government-led initiatives supported the growth of these technologies. The ORCHID (Organ-on-chip development) project was an initiative of the European Union (EU) to develop OOAC platforms, connect stakeholders, and bring innovative growth to the field (https://h2020-orchid.eu/) (accessed on 16 May 2022). Tissue Chips for Drug Screening is a project from the National Institutes of Health’s (NIH) National Centre for Advancing Translational Science (NCATS). It consists of different projects, such as developing tissue models or disease models, including innovative approaches such as growing tissue chips in microgravity. EVATAR, one of their popular projects, is a 3D OOAC platform of the liver and female reproductive tracts. This device can mimic the female reproductive system, including cyclical hormones. Although the liver is not a part of the female reproductive organ, it is involved in hormonal and drug metabolism associated with reproduction. This model can be used in studies related to fertility and women’s health. It can also be utilized to analyze hormonal and drug interactions, cervical cancer, endometriosis, and other issues related to the female reproductive system (https://ncats.nih.gov/tissuechip) (accessed on 16 May 2022). Some of the companies working on OOAC along with their products and a brief description of the features and limitations are listed in Table 3.

## 5. Applications of OOAC

### 5.1. Organ/Disease Modelling

In vitro modelling of disease pathways associated with several organs/organ systems has innumerable potential applications: analysis of organ anatomy, function, in-depth etiology of disease, the development of reliable diagnostics, and effective and well-tolerated therapeutic agents. Multiple organ models, associated diseases, and their purposes are listed in Table 4. X-linked retinitis pigmentosa is an inherited retinal dystrophy that results in premature blindness. R. Megaw et al. [109] developed a 3D retina organoid model to analyze the role of retinitis pigmentosa GTPase regulator (RGPR) in retinal degeneration. Ohlemacher and co-workers [110] engineered a model to investigate glaucoma through samples obtained from a genetically inheriting patient. This study further demonstrated that human pluripotent stem cells can be essential in pharmacological screening and disease modelling, which can narrow the gap caused by existing methods of clinical trials, testing and animal models, thereby allowing for the evaluation of disease progression with an in vitro system. Barth syndrome, an X-linked genetic disorder caused by a mutation in the *TAFAZZIN* gene, affects multiple organ systems. One of the major features of this disorder is cardiomyopathy, which usually occurs in the form of dilated cardiomyopathy and endocardial fibroelastosis. To study the pathophysiology of this disorder, Wang et al. [111] prepared an in vitro model of cardiomyopathy linked to the Barth Syndrome. iPSCs derived from unrelated individuals were used for the investigation. This model proved to be applicable for both drug discoveries and therapeutic purposes. An in vitro model of failing myocardium resulting from maladaptive cardiac hypertrophy was also developed and found to be useful in genetic and functional studies associated with the disease.

### 5.2. Pharmacology

A drug development process begins with early laboratory-based discovery stages and ends in the final marketing and surveillance once released to the industry. It involves five basic steps: (i) drug discovery and development; (ii) preclinical research; (iii) clinical development; (iv) FDA review and approval; and (v) safety monitoring post-release. This whole process takes a minimum of 10–15 years. If the investigated drug is not effective, incompatible with human metabolism, or has serious or fatal side effects, it results in substantial losses to both pharmaceutical and biotechnology companies [121,122]^,^. The rapid and accurate evaluation of drug efficiency as well as the impact of novel therapeutics on target sites and associated organs can be effectively monitored with OOAC models. In 2018, Seo and co-workers [123] successfully engineered a blinking human eye to assess cornea therapeutic drugs to ward off dry eye disease. With this experiment, they identified novel mechanobiology aspects of the ocular surface. Thrombosis is a common cause of death as it blocks blood vessels [124]. To estimate the efficacy of shear-targeted drugs, a replicating model of vascular stenosis was prepared. Nanoparticles were aggregated to administer while absorbing abnormal fluid shear stress [125]. This platform has potential applications in drug targeted delivery, drug screening, and development of novel thrombolytic drugs. Namdee and co-workers [126] prepared human microvessels to appraise the performance of vascular targeted carriers (VTC). The model demonstrated that nanospheres have a poor ability to localize the targeted vessel wall midstream. However, microspheres delivered better margination than nanospheres. These findings are of critical importance in drug screening.

### 5.3. Personalized Medicine

Similar classes of drugs are usually prescribed to multiple patients, depending on the disease being treated. In the clinical setting, drug efficacy and tolerability can be highly variable within the population. For instance, tolerance to Capecitabine, an oral chemotherapeutic commonly utilized in colorectal malignancies, is highly variable, such that a higher incidence of toxicities (neutropenia, gastrointestinal, and skin) and treatment discontinuation occurs in North American vs. East Asian populations [127].

We have merely scratched the surface of precision medicine. Genomic sequencing of solid tumors and identification of key molecular targets have changed the landscape of primary and metastatic tumors, from fatal diseases to chronic, potentially curable conditions.

The expansion of precision medicine into other diseases, such as HIV/AIDs, hepatitis B, and other chronic conditions, will revolutionize patient care. Precision medicine will allow for the development of highly efficacious and personalized therapy, based on a patient’s health history and genetic profile. Using health data and patient samples, the personalization of OOAC can be performed [128]. Sample acquisition can be easily obtained via blood, urine, stool, and/or biopsy samples. Benam and co-workers [129] formed a breathing airway-on-a-chip to study the pathophysiology of COPD and asthma. When the airway epithelium was exposed to a bacterial infection using polyinosinic:polycytidylic acid, a pro-inflammatory state was observed in the epithelium, mimicking the effects of asthma exacerbation. This device was effective in modelling patient-specific issues on a chip. Although primary tissue sample sources are minimal, the acquisition of patient-specific stem cells has several potential advantages in personalized medicine. Certain cell types, such as cardiomyocytes and podocytes, are difficult to regenerate on a chip; it is challenging to maintain differentiated cells in an in vitro environment, thus limiting the application of personalized medicine [130]. iPSCs have a high degree of differentiation and ability to sustain themselves in an in vitro environment; therefore, they can be used to overcome these constraints. To study the functional property of endothelial cells (ECs) derived from iPSCs, Halaidych and co-workers [131] experimented on mimicking the inflammatory response with endothelial-leukocyte interaction and analyzed barrier function with an assay of electric wound healing and real-time migration of EC. These are significant findings in studies focusing on patient-specific conditions.

### 5.4. Dentistry

According to a WHO report on 23 December 2020, more than 3.5 billion people suffer from oral diseases, a condition that has not improved from 1990 to 2017. Untreated dental caries in permanent teeth is the most dominant condition, affecting 2.3 billion people. Severe periodontal disease, a primary reason for tooth loss, is predicted to affect 267 million people, mainly older individuals. Cancers of the oral cavity are amongst the top 15 most common cancers worldwide, with over 500,000 cases and nearly 180,000 deaths per year [132].

The tooth has a unique biological configuration that is critical for disease treatment. Cells inside the dental pulp associate indirectly through a calcified permeable membrane, produced by the dentin matrix and numerous dentinal tubules of ∼2 μm in diameter. Though the cytotoxic response of the dental pulp to biomaterials has been studied widely, there is a lack of in vitro studies that recapitulate the dentin–pulp interface for understanding the morphologic, metabolic, and functional activity of biomaterials on live dental pulp cells. To address this topic, Cristaine and co-workers developed an organ-on-a-chip model system that links cells cultured on a dentin wall inside a microfluidic device that duplicates some of the structures and functions of the dentin–pulp interface. The tooth-on-a-chip is made of molded PDMS consisting of two chambers separated by a dentin fragment. To capture pulp cell responses to dental materials on-chip, stem cells from the apical papilla (SCAPs) were cultured in an odontogenic medium, seeded onto the dentin surface and observed under live-cell microscopy. Standard dental materials used clinically were tested for cytotoxicity, cell morphology, and metabolic activity on-chip and compared against standardized off-chip controls. In conclusion, the tooth-on-a-chip is a platform that mimics the physiologic conditions of the pulp–dentin interface and allows live-cell imaging to study dental pulp cell response to biomaterials [133].

Rahimi et al. [134] sought to develop an oral mucosa-on-a-chip with a lateral configuration consisting of undifferentiated keratinocytes and gingival fibroblasts to assess mucosal remodeling and the responses of epithelial and subepithelial layers to conditions typically found in the oral environment. A gingival fibroblast collagen hydrogel was gathered in the central channel of a three-channel microfluidic chamber with interconnecting pores, accompanied by a keratinocyte layer linked to the collagen exposed in the pores. Keratinocyte, fibroblast, and collagen densities were integrated to create a co-culture tissue-like construct stable over one week. Cells were stained and imaged with epi-fluorescence microscopy to confirm the layer characteristics. As a proof of concept, the mucosal construct was exposed separately to a dental monomer, 2-hydroxyethyl methacrylate (HEMA), and the oral bacteria *Streptococcus mutans*. Exposure to the dental monomer lessened mucosal cell viability, while exposure to the bacteria lowered trans-epithelial electrical resistance. These findings suggest that the oral mucosa-on-a-chip provides a suitable media for studying oral mucosal connections with bacteria and biomaterials through histology-like images of the tissue layers. Niu and co-workers [135] fabricated a microfluidic chip by soft lithography. The microchannels on the chip could replicate the microstructures of dentin tubules and the microchambers that link the microchannels, which could be used for odontoblast culture. The device successfully induced the growth of odontoblast processes from cell bodies; in addition, the use of various microchannel sizes (i.e., 2, 4, 6, and 8 μm) provided information on the relationship between the growth of odontoblast processes and the geometric constraint imposed by microchannels. The fabricated microfluidic chip can act as a powerful tool for future studies on the physiology and pathology of odontoblast process and the development of treatment solutions for dental diseases, such as dentin hypersensitivity.

## 6. Challenges and Future Perspective

Some of the OOAC technologies and inherent difficulties have been discussed in the previous sections. An in vitro system to model organs must be able to replicate the complete structural and functional mechanism of a specific organ type. However, none of the systems developed so far can replicate the entire physiology of an organ, prompting the search for more informative systems. PDMS is used for fabrication due to its excellent elastic properties; however, it has the limitation of shrinking upon absorbing some compounds [136]. Hence, alternative materials with improved performance need to be identified to scale up production before release to the industry. Sourcing of iPSCs and their reproducibility are additional limiting factors [137,138]. Since the fluidic system requires very precise extraction and injection of compounds, an automated fluid handling system is required [139]. Optical, electrical, and electrochemical sensors are used to monitor cellular and tissue responses; however, despite the advantage of enabling real-time analyses, these sensors are inefficient in handling small amounts of reagents in miniaturized dimensions [140]. Each of these sensors has their own advantages and disadvantages upon comparison with others. The choice of materials, chip position, and chip assembly play a major role in generating the proper output from the sensors. Saturation and regeneration are also obstacles associated with the current sensors. Development of a sensor-integrated OOAC platform relies on the development of other areas, such as microfabrication, material science, biosensors, and synthetic chemistry [141]. Microsensors with high sensitivity and specificity are required to measure physiological parameters more accurately and precisely. Since all of the human body systems are interconnected, the use of a single organ system on a chip would be desirable for the complete analysis of organ-specific mechanisms. Ultimately, to evaluate the whole system, i.e., the interaction between multiple organs, the mechanism of cell migration, and the process of cell signaling between them, a body-on-a-chip concept needs to be developed further.

Despite all these challenges, OOAC has a promising future and is an actively pursued field. Readers are encouraged to read another recent review on the subject [142]. Microbiorobotics (MBR) can be deployed in an OOAC for multiple purposes, such as drug delivery, real-time monitoring of the physiology of tissues in the cellular environment, and as a testbed [143]. Through these, a more precise and automated drug delivery to the target site is possible [144]. Other studies that integrate microsensors with OOAC could enable more reliable and accurate outputs from these experiments. Finally, new platforms should incorporate an essential mechanism to analyze the immune response when the system is subjected to foreign particles. With the emergence of new open-source code and better neural network models, the combination of OOAC and artificial intelligence can manage highly labor-intensive data, improving data analysis and automation. More AI integrated systems along with imaging modalities are expected to evolve in the near future.

## 7. Conclusions

Here we have discussed the development of various OOAC technologies, their fabrication techniques, some of the commercially available platforms, ongoing projects, the necessity of developing AI-integrated OOAC platforms and their applications. We have also mentioned data science technologies that are being integrated with OOAC to make it more reliable with improved functionality. It can be concluded that the technology is still in its developmental stage as most of the engineered models can only partially fulfill functions when considering an entire organ. However, step-by-step progress is noticeable as some developed models can exhibit breathing, peristalsis, and cardiac pacing. Developing a fully functional and efficient OOAC platform is contingent on many other developing areas, such as biomaterials, biofabrication, micro and biosensors, tissue engineering, and other cross-related areas. Since the human body is a complex system with highly complicated structural and functional mechanisms, it is not easy to replicate it in an in vitro environment, monitor the process, and analyze the data. Despite this, the research and development so far indicate that a wholly established and reliable system will be on the market soon. Applying biomedical, computational, and tissue engineering principles together can contribute a favorable outcome to the development of OOAC technologies. Governmental support will impact progress, as evident for some of the projects mentioned in the previous sections.

## Figures and Tables

**Table 1:** Lung-on-a-chip device fabrication techniques.

Devices	Fabrication Materials	Fabrication Techniques	Features	References
Pulmonary edema-on-a-chip	PDMS	Soft lithography	- Can replicate some of the physiological consequences of pulmonary edema - Reliable for drug efficacy and toxicity tests	[17]
Lung airway-on-a-chip	PMMA	Micromilling	- Open microfluidic device to suspend hydrogel - To study SMCs-ECs interaction	[18]
Airway-on-a-chip	PCL & PDMS	3D Bioprinting	- Formed an interface of vascular networks	[19]

**Table 2:** The advantages and disadvantages of the discussed organ-on-chip systems.

Type of OOAC	Advantages	Disadvantages
Lung-on-a-chip	Lung-on-a-chip is an excellent replacement to fill the gaps during the transition of test results from an in vitro model to an in vivo environment. One of the pioneering developments in the lung chip platform was the ability to replicate the breathing mechanism [17]. Second-generation lung-on-a-chip studies are aiming to replicate the alveolar network, including physical and biochemical characteristics of alveolar basal membrane by developing stretchable models [54].	Despite the advancements in lung-on-a-chip, the functional timeline of the chips last up to four weeks only, limiting modelling of chronic conditions. In addition, other characteristics such as cell-to-liquid ratio have to be addressed properly in order to avoid the dilution of metabolites, proteins, and other substances [54].
Heart-on-a-chip	The heart-on-a-chip system helps conduct studies on various cardiac diseases, drug screening and testing. Several associated platforms reviewed in this article have shown high throughput, portability, and the ability to replicate the cardiac system’s physical, electrical, biomechanical characteristics.	Since the heart is a complex structure, it is comparatively difficult to recreate an environment which consist of different types of cells with properties such as polarization and electric impulses to manage contraction of heart chambers, the alignment of these cells, and providing external stimuli. Efforts are being made to overcome this by adopting different fabricating techniques, such as 3D scaffolds and micropattern substrates [55].
Kidney-on-a-chip	The biomimetic kidney-on-a-chip has a significant role in drug toxicological and filtration studies. Various kidney chip models discussed in this article are being improvised at each stage and can retain highly relevant renal characteristics.	Some of the challenges in kidney-on-a-chip include occurrence of bubbles because of smaller dimensions of the chip, degradation of matrix, which can impact cell viability, and optimization of high throughput system. It is highly challenging to maintain consistency in cell seeding as it determines the chip’s characteristics. As mentioned above for other OOAC platforms, the viability and functionality may vary from 3 to 4 weeks [56].
Liver-on-a-chip	It is evident from the various studies that we reviewed on liver-on-a-chip, there is high reproducibility and can highly correlate chemical and toxicological testing. Some of the developed architecture of chip designs can replicate the in vivo physiological environment of the lung more closely.	Despite major advancements, there are still discrepancies in usage of cell sources. For example, stem cell induces hepatocytes and has a stable function, including albumin secretion and urea production. But they require specific induction factors and are costly. Primary hepatocytes can express liver intrinsic characteristics, but are difficult to isolate and incompatible in long term usage. Based on this, biomarker values vary along with discrepancies in the metabolic functions of these cells [57]. Liver chip platforms have low throughput which limits large scale industrial applications [58].

**Table 3:** Some of the leading OOAC platforms.

Company	System	Selected Products	Features	Limitations	Region	References
Mimetas	- PhaseGuide^TM^ technology, cells are free to interact and migrate, supporting cell-cell interaction, imaging, and quantification.	- OrganoPlate^®^ 2-lane 96 - OrganoPlate^®^ Graft - OrganoFlow^®^	- Layered tissue without artificial membranes - Automated imaging - Robotic liquid handling equipment	- Chips are not reusable after washing - Cell culture can be retained for up to 2 weeks only - OrganoPlates are non-compatible under electron microscopy	The Netherlands	[77,78,79,80]
Emulate	Human emulation system to culture multiple organs	- Brain chip - Kidney chip - Liver chip - Lung chip	- Stretch parameters to emulate peristalsis, breathing - Can culture up to 12 organ chips	- Chances of test material interaction with the chip can alter the output of the experiment [81]	USA	[82,83,84,85]
AxoSim	Nerve-on-a-chip	- NERVESIM^TM^ - BrainSIM^TM^	- Cultures iPSCs in a 3D environment	- Nerve conduction velocity for the developed platform is only about 0.13–0.28 m/s [86] - Limited automation on existing models	USA	[87,88]
TARA Biosystems	Heart-on-a-chip	- Biowire^TM^ II platform - Cardiotype	- Can develop disease models from patients	- Improvisation is needed to develop a closer physiologically biomimetic model [89]	USA	[90,91]
AlveoliX	Lung-on-a-chip	- ^AX^Lung-on-chip system	- Recreates air–blood barrier with ultra-thin membrane	- Since these utilize Collagen-Elastin (CE) membrane, the flexibility of the membrane depends on the ratio between both. - Gelation temperature has a direct impact on the mechanical properties of the membrane [54,92]	Switzerland	[93,94]
TissUse	Human-on-a-chip	- HUMIMIC Chip 2 - HUMIMIC Chip 3 - HUMIMIC Chip 4 - HUMIMIC Chip XX/XY	- Can mimic biological barriers while integrating multiple organs on a chip - Long term performance	- Single-use devices. - Chips can be stored only for 7 days. - For longer use, the buffer solution must be changed, which voids guarantee.	Germany	[95,96,97,98]
CN Bio Innovations	- Single organ-on-a-chip - Multiple organs-on-a-chip	- PhysioMimix^TM^ - Liver-on-a-chip (MPS-LC12)	- Recirculating fluid flow to deliver essential materials - Inter- and intra-organ-specific flow rate can be adjusted	- A high-level system still has to be developed to replicate multi-organs-on-a-chip to mimic all the physiological function of organ systems [99]	UK	[100]
Kirkstall	- QuasiVivo^®^, an interconnected cell culture flow system for growth of cell	- QV500 - QV600 - QV900	- They are flexible and long-term culture is possible	- The chambers are made of PDMS and there are chances of components getting absorbed, which can alter experimental outcomes.	UK	[101,102]
SynVivo	3D tissue and OOAC model	- SynTumour 3D Cancer model - SynALI Lung model - SynBBB Blood-Brain Barrier model	- Quantitative real-time visualization is possible	- Low throughput system - Require improvised design for the chip to enhance the seeding capacity [103]	USA	[104]
Hesperos Inc.	- Multi-organ micro physiological system	- Heart-liver two organ model - Neuromuscular junction two organ model	- Uses a serum-free cell medium - Posse’s gravity flow system	- Studies are conducted in monoculture and co-cultures studies need to be conducted for reliability [105].	USA	[105]
InSphero	- Organ-on-a-chip system - Production of microtissues using 3D Select^TM^ process	- 3D Insight tumor microtissues - 3D Insight islet microtissues - 3D Insight liver microtissues	- Can capture long term drug effect - In vivo-like morphology and functionality	- Further studies have to be conducted on human iPSCs to understand predictive power of assays [106]	Switzerland	[106,107]
Nortis Bio	- Organ-on-a-chip - Perfusion system platform	- ParVivo Chips	- Vascularization of tissues - Produce tumor microenvironment	- ParVivo Chips have 96-well plate footprint and are 2 inches high, limiting their compatibility with specialized microscopes.	USA	[108]

**Table 4:** Some examples of OOAC technology used for organ/disease modelling.

Organs	Devices & Their Purposes	References
Eye	Age-related macular degeneration model to replicate mechanical stress on retinal pigment epithelial cells	[112]
Heart	Heart-on-a-chip platform to analyze hypoxia-induced myocardial injury by using cyanide-p-trifluoromethoxyphenylhydrazone to block oxygen consumption	[113]
Vasculature	Microfluidic model to study clot formation useful in the analysis of thrombosis and angiogenesis	[114]
Kidney	Model of biomimetic glomerulus-on-a-chip and diabetic kidney to study diabetic nephropathy	[115]
Lung	Human airway-on-a-chip was prepared using mucociliary bronchiolar epithelium, which is infected with human rhinovirus to study factors causing asthma	[116]
Gut	Human gut-on-a-chip to study gut-immune interactions	[117]
Liver	Organoids-on-a-chip using iPSCs to model NAFLD	[118]
Bone	In vitro model micro vascularized bone to study the interaction between cell and bone matrix	[119]
Brain	Epileptic seizure model of the brain from pluripotent stem cells with the ability to mimic local and circuitry function of brain	[120]

## Data Availability

Not applicable.
